# Differentiated responses of the phyllosphere bacterial community of the yellowhorn tree to precipitation and temperature regimes across Northern China

**DOI:** 10.3389/fpls.2023.1265362

**Published:** 2023-10-25

**Authors:** Weixiong Wang, Congcong Hu, Yu Chang, Libing Wang, Quanxin Bi, Xin Lu, Zhimin Zheng, Xiaoqi Zheng, Di Wu, Ben Niu

**Affiliations:** ^1^ State Key Laboratory of Tree Genetics and Breeding, Northeast Forestry University, Harbin, China; ^2^ The Center for Basic Forestry Research, College of Forestry, Northeast Forestry University, Harbin, China; ^3^ College of Life Science, Northeast Forestry University, Harbin, China; ^4^ Department of Mathematics, Shanghai Normal University, Shanghai, China; ^5^ State Key Laboratory of Tree Genetics and Breeding, Research Institute of Forestry, Chinese Academy of Forestry, Beijing, China; ^6^ Chifeng Research Institute of Forestry Science, Chifeng, China; ^7^ National Forestry and Grassland Shiny-Leaved Yellowhorn Engineering and Technology Research Center, Chifeng, China; ^8^ Center for Single-Cell Omics, School of Public Health, Shanghai Jiao Tong University, School of Medicine, Shanghai, China

**Keywords:** yellowhorn, phyllosphere bacterial community, climate factors, community composition, microbial networks, keystone taxa

## Abstract

**Introduction:**

As an ephemeral and oligotrophic environment, the phyllosphere harbors many highly diverse microorganisms. Importantly, it is known that their colonization of plant leaf surfaces is considerably influenced by a few abiotic factors related to climatic conditions. Yet how the dynamics of phyllosphere bacterial community assembly are shaped by detailed climatological elements, such as various bioclimatic variables, remains poorly understood.

**Methods:**

Using high-throughput 16S rRNA gene amplicon sequencing technology, we analyzed the bacterial communities inhabiting the leaf surfaces of an oilseed tree, yellowhorn (*Xanthoceras sorbifolium*), grown at four sites (Yinchuan, Otogqianqi, Tongliao, and Zhangwu) whose climatic status differs in northern China.

**Results and Discussion:**

We found that the yellowhorn phyllosphere’s bacterial community was generally dominated by four phyla: Proteobacteria, Firmicutes, Actinobacteria, and Bacteroidetes. Nevertheless, bacterial community composition differed significantly among the four sampled site regions, indicating the possible impact of climatological factors upon the phyllosphere microbiome. Interestingly, we also noted that the α-diversities of phyllosphere microbiota showed strong positive or negative correlation with 13 bioclimatic factors (including 7 precipitation factors and 6 temperature factors). Furthermore, the relative abundances of 55 amplicon sequence variants (ASVs), including three ASVs representing two keystone taxa (the genera *Curtobacterium* and *Streptomyces*), exhibited significant yet contrary responses to the precipitation and temperature climatic variables. That pattern was consistent with all ASVs’ trends of possessing opposite correlations to those two parameter classes. In addition, the total number of links and nodes, which conveys community network complexity, increased with rising values of most temperature variables. Besides that, remarkably positive relevance was found between average clustering coefficient and most precipitation variables. Altogether, these results suggest the yellowhorn phyllosphere bacterial community is capable of responding to variation in rainfall and temperature regimes in distinctive ways.

## 1 Introduction

Phyllosphere, which consists of the aerial surface area portion of plants, is one of the most prevalent and important habitats for diverse microorganisms, including bacteria, fungi, archaea, as well as viruses (Vorholt, 2012; Muller and Ruppel, 2014; Sohrabi et al., 2023), of which bacteria are numerically dominant in the phyllosphere (Martirosyan et al., 2016). These phyllosphere microorganisms, which colonize at the plant–climate interface(Liu et al., 2023b), provide their hosts with a diverse range of beneficial metabolic and functional effects (Sohrabi et al., 2023). For example, they were reported to promote the plant growth and development, to augment plant resistance to pathogens, and to neutralize harmful chemicals, as well as to facilitate adjustments for climatic change (e.g., temperature, air humidity, and rain), all of which play an essential role in fostering host plants’ health and their ecological strategies (Vorholt, 2012; Li et al., 2019; Singh et al., 2019; Li et al., 2022b). Thus, as a unique microhabitat in foliage, the phyllosphere harbors diverse microbial communities that may be key contributors to improving the productivity and stress resistance of plants (Liu et al., 2023a; Sohrabi et al., 2023).

Climate is one of the major factors that influences the distribution and composition of microbial communities in nature (Vacher et al., 2016; Zhu and Penuelas, 2020; Faticov et al., 2021). Different microbial species have different tolerance levels to moisture and temperature in the atmospheric environment, and the phyllosphere is always under direct exposure to the atmospheric environment. Accordingly, phyllosphere microbial communities could be undergo pronounced shifts in response to one or more climatic stresses (Compant et al., 2010; Singh et al., 2010; Penuelas et al., 2012; Singh et al., 2018). It is known that several phyllosphere microbiomes do show large fluctuations in the abundance of their populations before and after rainfall events (Hirano et al., 1996; Pietrarelli et al., 2006). For small or low-abundance population in particular, such phyllosphere microbial species dwelling on the leaf surface are more likely to be removed from the host during the process of rainfall (Pietrarelli et al., 2006; Stone and Jackson, 2019). Another pertinent factor is the rising atmospheric temperature, which can also alter the diversity, composition, and activities of the microbial community in phyllosphere, since higher temperatures could aggravate various positive or negative interactions among microbes by accelerating individual metabolic processes and growth (Xue et al., 2016; Yuan et al., 2021). However, recent studies on the impact of precipitation and air temperature on the structure of phyllosphere microbial communities mainly focus on seasonal variation (Howe et al., 2023), long-term warming (Aydogan et al., 2018), or distinct climatic zones (Firrincieli et al., 2020). Yet changes in the composition and network characteristics of the epiphytic bacterial community associated with the phyllosphere in response to multiple precipitation and temperature variables in tandem are still poorly understood. Thus, it is imperative to elucidate how the phyllosphere bacterial community adapts to climatic variation, which is of great ecological importance for robustly dissecting their roles in improving plant growth and fitness.

Yellowhorn (*Xanthoceras sorbifolium*) is an oil woody plant native to China (Bi et al., 2019). It is widely distributed across China’s northern regions due to its superior tolerance of drought, cold, salt, and alkali stress conditions (Yu et al., 2012; Guo et al., 2013; Sun et al., 2018; Bi et al., 2019). Accordingly, yellowhorn offers tremendous multifaceted benefits in terms of both ecological and economic value, as well as high potential medicinal value (Liu et al., 2013; Wang et al., 2017; Li et al., 2020). Although the phyllosphere microbiome of other plants in response to seasonal changes or climate types has been reported (Bao et al., 2020; Firrincieli et al., 2020), our understanding of the climatic factors-driven bacterial community assembly of yellowhorn phyllosphere is still limited. Understanding the climatic drivers of the phyllosphere bacterial community composition of yellowhorn underlying the protection of yellowhorn against climatic stresses by phyllosphere microorganisms.

To that end, in this study, we selected yellowhorn from four typical regions (Yinchuan, Otogqianqi, Tongliao, and Zhangwu) in northern China. Our goals in this study are three-fold: (1) to characterize the composition and distribution pattern of phyllosphere bacterial communities of yellowhorn from different regions; (2) to determine the potential influence of climatic factors on the diversity, composition, assembly, and key functional taxa of those ​phyllosphere bacterial communities; (3) to reveal the topological properties and network stability within the phyllosphere bacterial community of yellowhorn and the ecological niches of keystone taxa. This correlative research into climate factors vis-à-vis characteristics and network features of phyllosphere bacterial communities of yellowhorn should advance our understanding the role played by climate in microbiome assembly on plant leaf surfaces. This knowledge could therefore serve as a scientific basis for augmenting the environmental adaptability of yellowhorn.

## 2 Materials and methods

### 2.1 Study sites description and sampling

The study sites were located in Yinchuan City (YC, 106.17°E, 38.42°N), Otogqianqi City (OQ, 107.43°E, 38.18°N), Tongliao City (TL, 122.28°E, 43.63°N), and Zhangwu City (ZW, 122.52°E, 42.38°N) in northern China. The climatic data for these four site regions, e.g., annual mean temperature, mean diurnal range, isothermality, min temperature of coldest month, mean temperature of driest quarter, etc., are provided in **Supplementary Table 1**. The climatic data [The script for downloading the values of the bioclimatic variables from WorldClim database is available in GitHub (https://github.com/Tree-microbiome)] of four site regions was obtained from the Worldclim database (www.worldclim.org). The leaf samples of yellowhorn (*Xanthoceras sorbifolium* ‘Zhongshi 9’) were collected in July 2019 from the Yinchuan, Otogqianqi, Tongliao, and Zhangwu sites. At each site, 10 individual trees of yellowhorn were randomly selected. Ten pinnate compound leaves from the crown of each tree were collected and packed in a sterile plastic Ziplock bag. These collected leaf samples were then quickly taken to the laboratory for 16S rRNA sequencing and downstream data analysis of their phyllosphere bacterial community.

### 2.2 DNA extraction, PCR amplification, and sequencing

For the acquisition of the phyllosphere bacterial community, the surface of each leaf sample was wiped down with sterile cotton swabs containing 0.1% Tween 20 (Cregger et al., 2018). The phyllosphere bacterial community of leaf samples from the same tree were collected and then were mixed into one sample for the extraction of phyllosphere microbial genomic DNA (n = 10 samples per site). These cotton swabs were placed in a 1.5-mL centrifuge tube and quickly frozen with liquid nitrogen. All tubes containing cotton swabs were preserved at −80°C until their DNA extraction stop. The genomic DNA of the phyllosphere bacterial community at the tree level was extracted using a DNeasy Power Soil kit (QIAGEN, Hilden, Germany) according to the manufacturer’s instructions.

The V4 region of the bacterial 16S rRNA gene from these DNA samples was amplified with the primers 515F (5′-GTGCCAGCMGCCGCGGTAA-3′) and 806R (5′-GGACTACHVGGGTWTCTAAT-3′). Each sample was amplified in triplicate, in a 25-μL reaction system which contained 1.0 μL of genomic DNA, 12 μL of PCR water (QIAGEN, Hilden, Germany), 10 μL of 5 Prime Hot Master Mix (Quantabio, Beverly, USA), and 1.0 μL of each forward/reverse primers (10 nM final concentration). For each PCR amplification, the reaction was held at 94°C for 3 min, followed by 35 cycles at 94°C for 45 s, 50°C for 60 s, and 72°C for 90 s, and with a final extension for 10 min at 72°C. Next, three PCR amplicon products from the same microbial genomic DNA were combined into one sample and then were quantified using a Quant-iT dsDNA Assay Kit (invitrogen, Oregon, USA). 200 ng DNA of each sample was taken to establish the amplicon library. This amplicon library was purified using the QIAquick Gel Extraction Kit (QIAGEN, Hilden, Germany) and was sequenced on Illumina MiSeq platform by the Shanghai Personal Biotechnology Co., Ltd. (Shanghai, China).

### 2.3 Sequence data processing

The QIIME2 (2020.2) platform was used to process the obtained sequencing data, (Bolyen et al., 2019). In detail, sequence quality control, including de-noising, trimming, and chimera removal was carried out using the DADA2 plugin, with amplicon sequence variants (ASVs) as the output (Callahan et al., 2016). Taxonomic classification of ASVs was carried out by using the QIIME2-feature classifier, based on 99% or greater sequence identity with the Greengenes database (v13.8) (McDonald et al., 2012) and SILVA database (v138). Sequences classified by SILVA database were used for the identification of the unique plus shared ASVs and the analysis of the composition of the shared ASVs.

### 2.4 Statistical analyses

All statistical analyses were performed in R computing platform (v4.0.4). Bubble graphs were drawn using the ‘igraph’ R package, in which the bubble size was proportional to the relative abundance of the read counts. The Shannon diversity, observed species, evenness, and Faith’s phylogenetic diversity (Faith_pd) of each microbial community were calculated to evaluate its alpha-diversity. Four indices were calculated in QIIME2 (v2020.2) and visualized in boxplots by using the ‘ggplot2’ and ‘ggpubr’ packages in R. Principal co-ordinate analysis (PCoA) was conducted to assess the variation between different groups based on Bray–Curtis dissimilarity matrices. To evaluate the variance in the phyllosphere bacterial community, permutational multivariate analysis of variance (PERMANOVA) was implemented using the “adonis” function in the ‘vegan’ package.

Significant differences at phyla or genera level were performed using STAMP software (ANOVA and Tukey–Kramer were used as statistical test and *post-hoc* test at a *P*-value of 0.05, respectively). The linear discriminant analysis effect size (LEfSe) was used to identify the biomarker taxa for each site by using the online tool at http://huttenhower.sph.harvard.edu/galaxy/ (the LDA threshold score of 2.0) (Li et al., 2022a). The correlation matrix was calculated by the ‘Hmisc’ package, where Spearman rank correlation was used to gauge the relationships between climate factors and the relative abundance of ASVs, α-diversity indices and network topological attributes. The co-occurrence network of the ASVs was constructed using the Molecular Ecology Network Analysis pipeline (http://ieg4.rccc.ou.edu/MENA/) under random matrix theory (RMT). The networks were visualized using Gephi software (v0.9.2).

## 3 Results

### 3.1 Composition of the phyllosphere bacterial community of yellowhorn

To investigate how climatic factors, for example, temperature and precipitation, impact the assembly of bacterial phyllosphere microbiome of yellowhorn (*Xanthoceras sorbifolium* ‘Zhongshi 9’), we selected four sampling sites in its main planting area in China, which are located at Yinchuan, Otogqianqi, Tongliao and Zhangwu, respectively. Specifically, the first two site regions, Yinchuan and Otogqianqi are situated in the northwest region of China, while the other two, Tongliao and Zhangwu are located in the northeast (**Figure S1**). The long distances between the northwestern sampling sites and the northeastern ones (1,300 to 1,500 kilometers) and the remarkable difference in altitudes (northwestern sites: 1,100 to 1,300 meters; northeastern sites: 85 to 180 meters) lead to the significantly differentiated climatic characteristics for these two sampling site groups (northwestern sites: semi-humid and semi-arid continental climate; northeastern sites: semi-arid continental monsoon climate)(Li et al., 2016; Li et al., 2018; Zhang et al., 2018; Guo, 2022) (**Supplementary Table 1**). Then, leaf samples of yellowhorn were collected in July 2019 from the above four sites, which were subjected to genomic DNA extraction in laboratory and subsequent 16S rRNA gene amplicon sequencing analysis.

**Figure 1:**
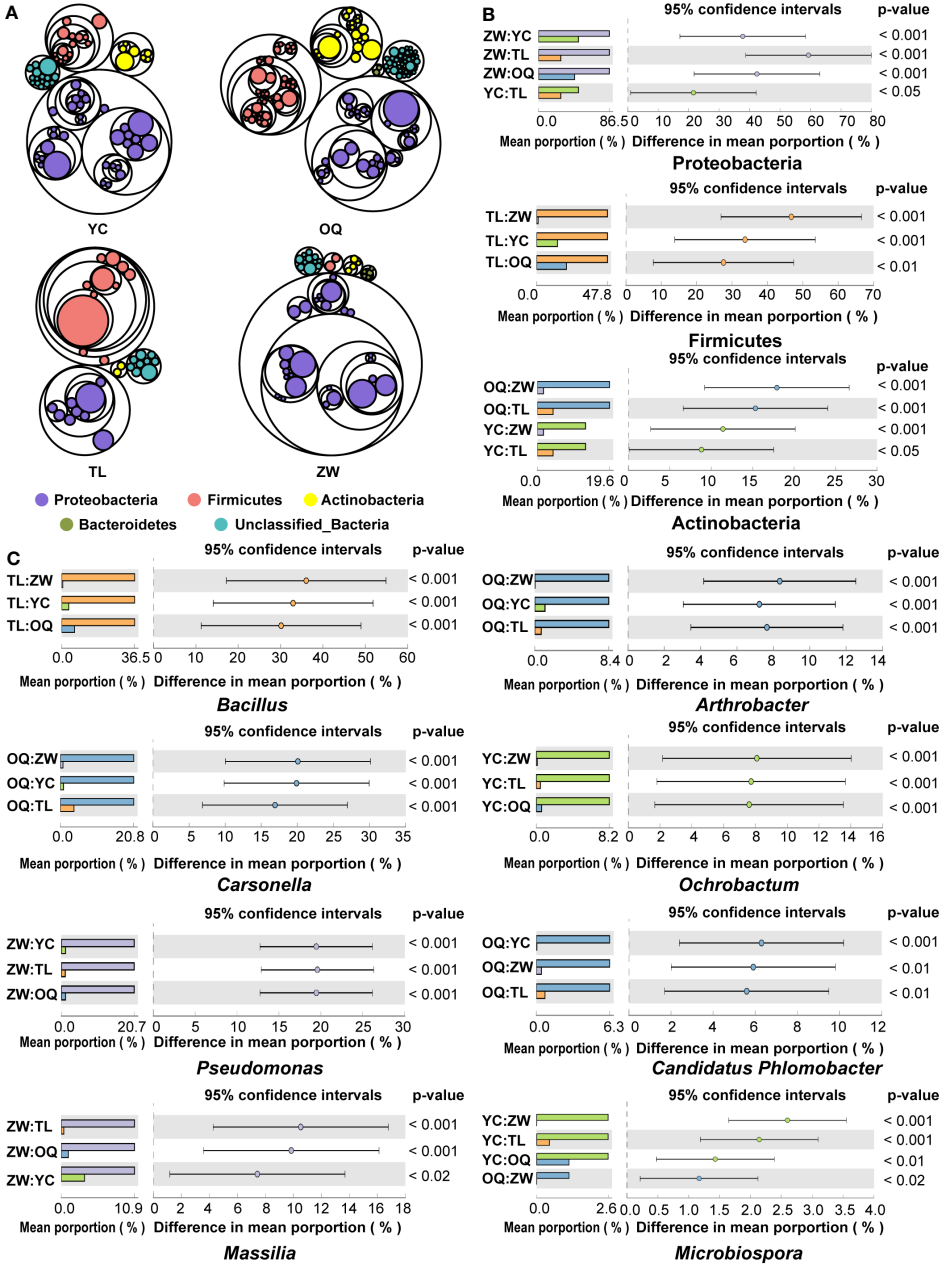
Compositions of the yellowhorn phyllosphere bacterial communities. **(A)** Compositions of phyllosphere bacterial communities of yellowhorn grown in the four sampling sites across north China. Circles from outside to inside sequentially represent the phylum, class, family, and genus. The size of circles is proportional to the relative abundance of each taxon, with those having the same color indicating the taxon belonging to the same phylum. **(B)** Relative abundance of the dominant phylum in the phyllosphere bacterial communities of the yellowhorn grown in the four site regions (ANOVA and Tukey–Kramer were used as statistical test and *post-hoc* test at a *P*-value of 0.05, respectively). **(C)** Relative abundance of the dominant genera in the phyllosphere bacterial communities of the yellowhorn grown in the four site regions (ANOVA and Tukey–Kramer were used as statistical test and *post-hoc* test at a *P*-value of 0.05, respectively). YC, Yinchuan; OQ, Otogqianqi; TL, Tongliao; ZW, Zhangwu.

Across all the samples, we obtained a total of 546,251 high-quality reads, with an average of 13,656 reads per sample. The high-quality reads were clustered into 5,158 ASVs according to ≥ 99% sequence identity as the cutoff (**Supplementary Table 2**). The identified sequences of the phyllosphere bacterial community of yellowhorn trees in different site regions were affiliated with 19 bacterial phyla. Of these phyla, Proteobacteria, Firmicutes, Actinobacteria, and Bacteroidetes were predominant, together accounting for more than 83.20% of the total reads (**Figure 1A**; **Supplementary Table 3**). Comparative analyses were applied to reveal distinctions in the predominant bacterial phyla. Notably, a significantly higher abundance of the phylum Proteobacteria but a significantly lower abundance of the phylum Firmicutes characterized the phyllosphere bacterial community from Zhangwu, while the phyllosphere bacterial community in Tongliao had a lower abundance of the phylum Proteobacteria yet a higher abundance of the phylum Firmicutes (**Figure 1B**). A greater distribution of the phylum Actinobacteria was found in Otogqianqi, whereas the distribution of the phylum Bacteroidetes was similar among all four site regions sampled.

At the genus level, the high-quality bacterial sequences from all samples were assigned to 390 genera. Of these, *Bacillus*, *Carsonella*, *Pseudomonas*, *Massilia*, *Acinetobacter*, *Arthrobacter*, *Ochrobactrum*, *Candidatus.Phlomobacter*, *Paenibacillus*, *Staphylococcus*, *Microbispora*, and *Curtobacterium* were the most abundant genera present in all samples (defined here as having an average relative abundance > 1%) (**Supplementary Table 4**). Evidently, these dominant genera featured pronounced regional distribution characteristics (**Figure 1C**). The genus *Bacillus* constituted the highest proportion of ASVs at Tongliao, while at Otogqianqi the following bacterial genera had higher relative abundances: *Arthrobacter*, *Carsonella*, and *Candidatus.Phlomobacter*. Further, the relative abundances of the genera *Pseudomonas* and *Massilia* were significantly higher at Zhangwu than the other three site regions, while the *Ochrobactrum* and *Microbispora* genera were most commonly found at Yinchuan. Across the four site regions, there was no significant difference in the distribution of *Acinetobacter*, *Paenibacillus*, *Staphylococcus*, or *Curtobacterium*.

**Figure 2:**
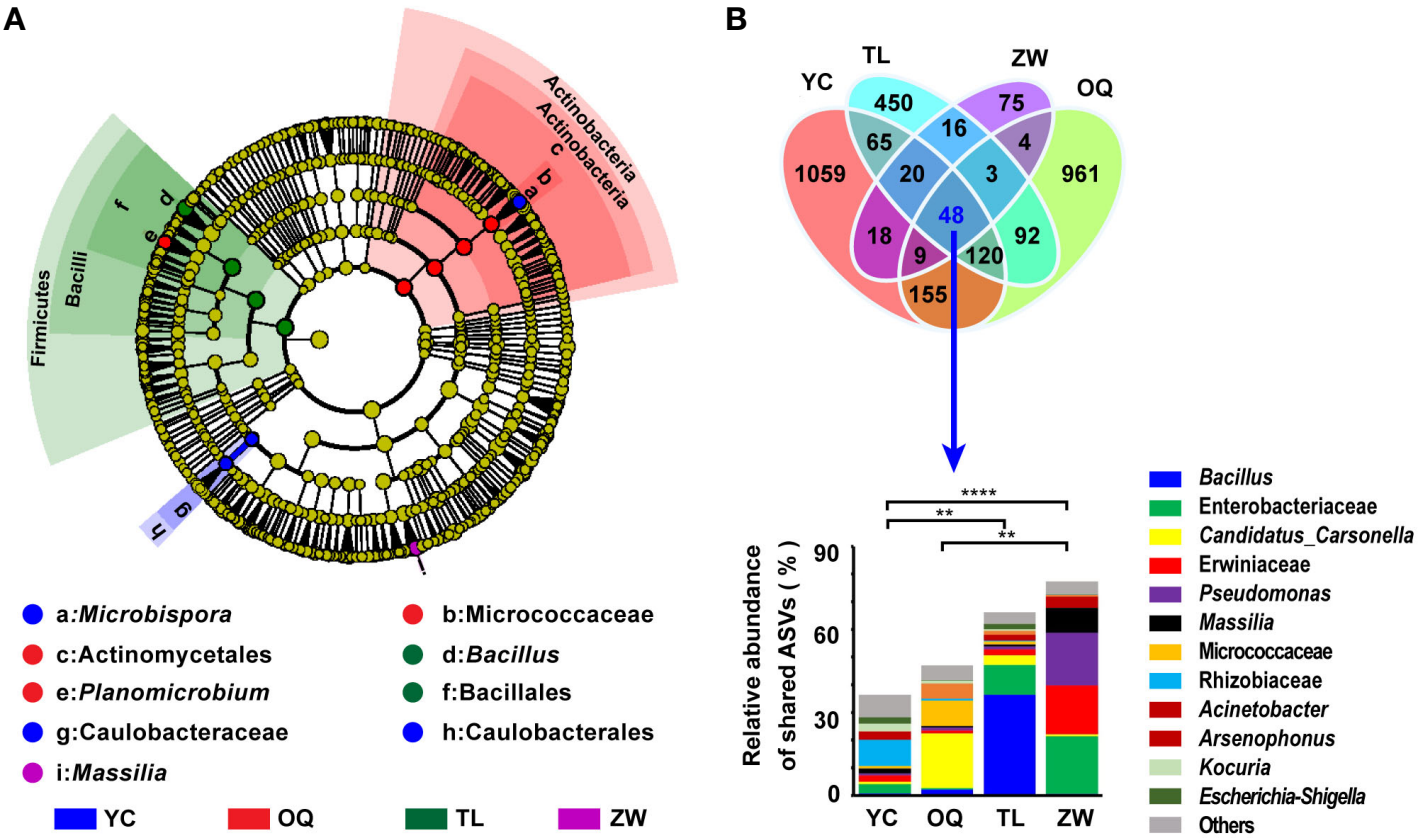
Taxonomic cladogram of yellowhorn phyllosphere bacterial communities and compositions of the shared amplicon sequence variants (ASVs). **(A)** Taxonomic cladogram of the phyllosphere bacterial communities of yellowhorn grown in the four site regions obtained by the linear discriminant analysis effect size (LefSe) analysis (LDA threshold of 2.0). **(B)** Venn diagram for the unique and shared ASVs in the phyllosphere bacterial communities of yellowhorn grown in the four site regions and relative abundance of these shared ASVs at the genus level. YC, Yinchuan; OQ, Otogqianqi; TL, Tongliao; ZW, Zhangwu. Asterisks indicate that differences among the means represented by the columns are statistically significant (^**^ *P* < 0.01; ^****^ *P*< 0.0001). *t* test was used for the analysis.

The taxa differences in the phyllosphere bacterial composition structure of yellowhorn were detected between the different regions from phylum to genus levels. **Figure 2A** shows the cladogram for the composition of the predominant discriminating bacteria among different site regions. The LEfSe analysis detected 13 discriminative taxa (**Figure 2A**). Members of bacterial taxa in the phylum Firmicutes were enriched in the phyllosphere samples from Tongliao, while those in the Actinobacteria phylum were enriched more in phyllosphere samples from the Otogqianqi site region. The order Caulobacterales, family Caulobacteraceae, and genus *Microbispora* were significantly more abundant in samples from Yinchuan. The genus *Massilia* could be used as a biomarker to discriminate the phyllosphere samples from Zhangwu (**Figure 2A**). Overall, the indicator taxa in each site region were largely consistent with its relative distribution of dominant genera there.

A Venn diagram was drawn to depict the shared and unique ASVs among samples from the four site regions. Only 48 ASVs of the phyllosphere bacterial communities were shared across the four sites, mainly assigned to these five phyla: Proteobacteria, Firmicutes, Actinobacteria, Bacteroidetes, and Myxococcota. These shared ASVs constituted 36.41%, 46.97%, 66.21%, and 77.29% of the total reads in the Yinchuan, Otogqianqi, Tongliao, and Zhangwu samples, respectively (**Figure 2B**). In stark contrast, the number of unique ASVs detected from these phyllosphere samples of Yinchuan, Otogqianqi, Tongliao, and Zhangwu site regions amounted to 1059, 961, 450, and 75, respectively (**Figure 2B**). Generally, more unique ASVs were present in Yinchuan, whereas a higher relative abundance of shared ASVs was found in Zhangwu.

### 3.2 Association of phyllosphere bacterial community structure with climatic variables

**Figure 3:**
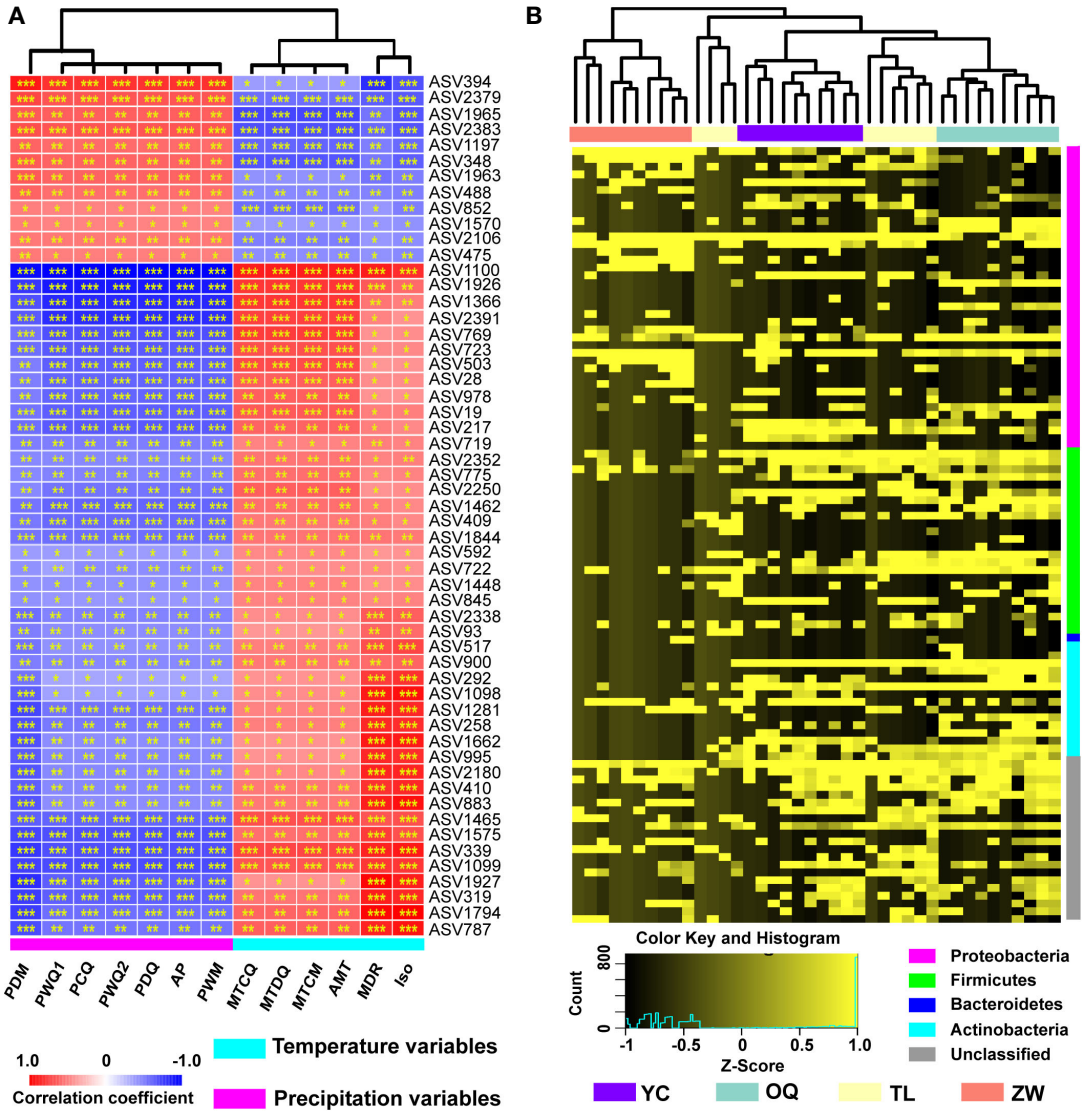
Correlation of climatic variables with relative abundance of amplicon sequence variants (ASVs) and hierarchical cluster analysis of the yellowhorn phyllosphere bacterial communities. **(A)** The red color denotes a positive correlation, while the blue color represents a negative correlation. The values of correlation coefficient range from -1 (blue) to 1 (red). Asterisks indicate significant relevance between the ASVs and the climatic variables (*P < 0.05; **P < 0.01; ***P < 0.001). AMT, annual mean temperature; MDR, mean diurnal range; Iso, isothermality; MTCM, min temperature of coldest month; MTDQ, mean temperature of driest quarter; MTCQ, mean temperature of coldest quarter; AP, annual precipitation; PWM, precipitation of wettest month; PDM, precipitation of driest month; PWQ1, precipitation of wettest quarter; PDQ, precipitation of driest quarter; PWQ2, precipitation of warmest quarter; PCQ, precipitation of coldest quarter. **(B)** The abundance of top 100 ASVs normalized by Z-Score scaling was analyzed by calculating Bray-Curtis dissimilarity matrix. The normalized ASV abundance ranges from low (black) to high (yellow). YC, Yinchuan; OQ, Otogqianqi; TL, Tongliao; ZW, Zhangwu.

Climatic variables also had an important impact upon the assembly of phyllosphere bacterial communities. According to the Spearman correlation, all ASVs of the phyllosphere bacterial community of yellowhorn were positively or negatively correlated with climatic variables, with 55 ASVs being significantly correlated with all 13 climatic variables examined (**Figure 3A**; **Supplementary Table 5**). Of those 55 ASVs, 12 of them—ASV394, ASV2379, ASV1965, ASV2383, ASV1197, ASV348, ASV1963, ASV488, ASV852, ASV1570, ASV2106, and ASV475—were positively correlated with precipitation variables but negatively correlated with temperature variables. The opposite trend was observed for all ASVs vis-à-vis the same climatic variables (**Figure 3A**).

### 3.3 Phyllosphere bacterial community diversity of yellowhorn

To analyze the dissimilarities among the yellowhorn phyllosphere bacterial communities across the four sampling sites, cluster analysis of top 100 ASVs was applied by calculating the Bray-Curtis dissimilarity distance (**Figure 3B**). The results show that these bacterial communities from Yinchuan, Otogqianqi and Zhangwu are clustered into three independent branches, indicating that the compositions of the phyllosphere bacterial communities of yellowhorn grown in these three regions are different from each other (**Figure 3B**).

**Figure 4:**
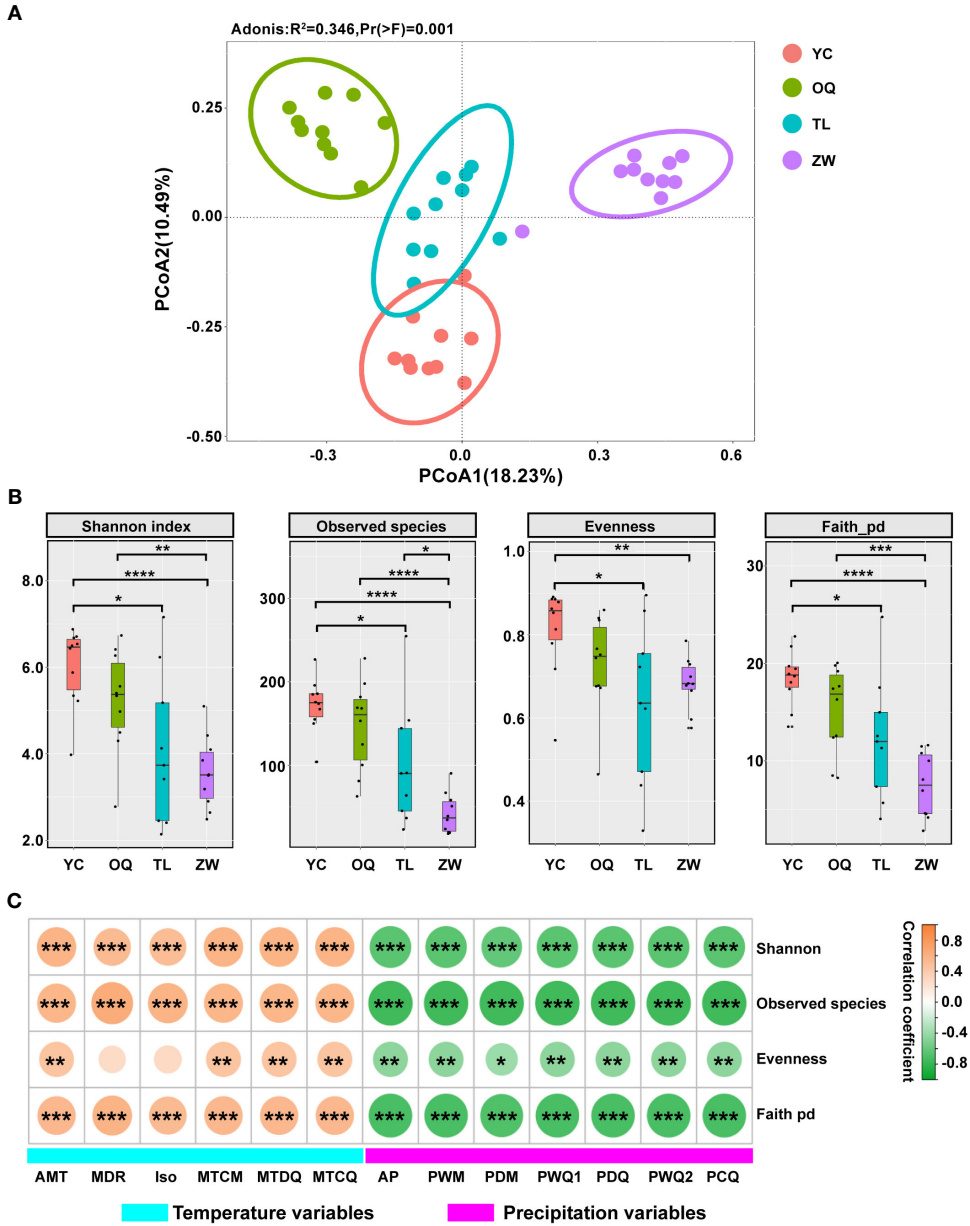
Alpha and beta diversity and correlation analysis of the phyllosphere microbial communities of yellowhorn. **(A)** Principal co-ordinates analysis (PCoA) of the phyllosphere microbial communities of yellowhorn among the Yinchuan (YC), Otogqianqi (OQ), Tongliao (TL), and Zhangwu (ZW) site regions (n = 10 per location) in Northern China. **(B)** Alpha diversity indices and significant difference analysis of phyllosphere bacterial communities of yellowhorn among the different regions according to the *t* test (*P< 0.05; **P < 0.01; ***P < 0.001; ****P < 0.0001). **(C)** Correlations between α-diversity indices and climatic variables. The orange color denotes a positive correlation, while the green color denotes a negative correlation. Asterisks indicate significant correlations between α-diversity indices and climatic variables (*P< 0.05; **P< 0.01; ***P< 0.001).

To further verify the results from cluster analysis, the PCoA was used to further evaluate the dissimilarity of phyllosphere bacterial community among samples of yellowhorn. The first two principal components together explained 28.72% of bacterial community variance. The phyllosphere bacterial communities from Yinchuan, Otogqianqi, Tongliao, and Zhangwu site regions separated neatly into four groups in the PCoA plot. This demonstrated that stronger discrepancies between bacterial communities could arise from different regions (**Figure 4A**). The adonis analysis also indicated a significant difference among the four groups (r^2^ = 0.346, *P* = 0.001).

Four α-diversity indices, observed species (richness), Shannon index (diversity), evenness, and Faith_pd (community phylogenetic diversity) were used to assess the divergence of phyllosphere bacterial communities from different site regions (**Figure 4B**). Across them, all four α-diversity indices of the phyllosphere bacterial community were estimated to be lower in Zhangwu than those in other site regions. These indices from Tongliao were much lower than those from Yinchuan, with no significant differences found between Yinchuan and Otogqianqi, or between Tongliao and Zhangwu.

We next examined the relationships of the four α-diversity indices to six temperature-related variables (annual mean temperature, mean diurnal range, isothermality, min temperature of coldest month, mean temperature of driest quarter, and mean temperature of coldest quarter) and seven precipitation variables (annual precipitation, precipitation of wettest month, precipitation of driest month, precipitation of wettest quarter, precipitation of driest quarter, precipitation of warmest quarter, and precipitation of coldest quarter) (**Figure 4C**; **Supplementary Table 6**). Notably, all four α-diversity indices showed strong positively correlation with temperature variables, except for evenness with mean diurnal range and isothermality (which showed only week correlation) (**Figure 4C**). In contrast, all the precipitation variables were significantly negatively correlated with each index (**Figure 4C**).

### 3.4 Molecular ecological patterns of phyllosphere bacterial community

**Figure 5:**
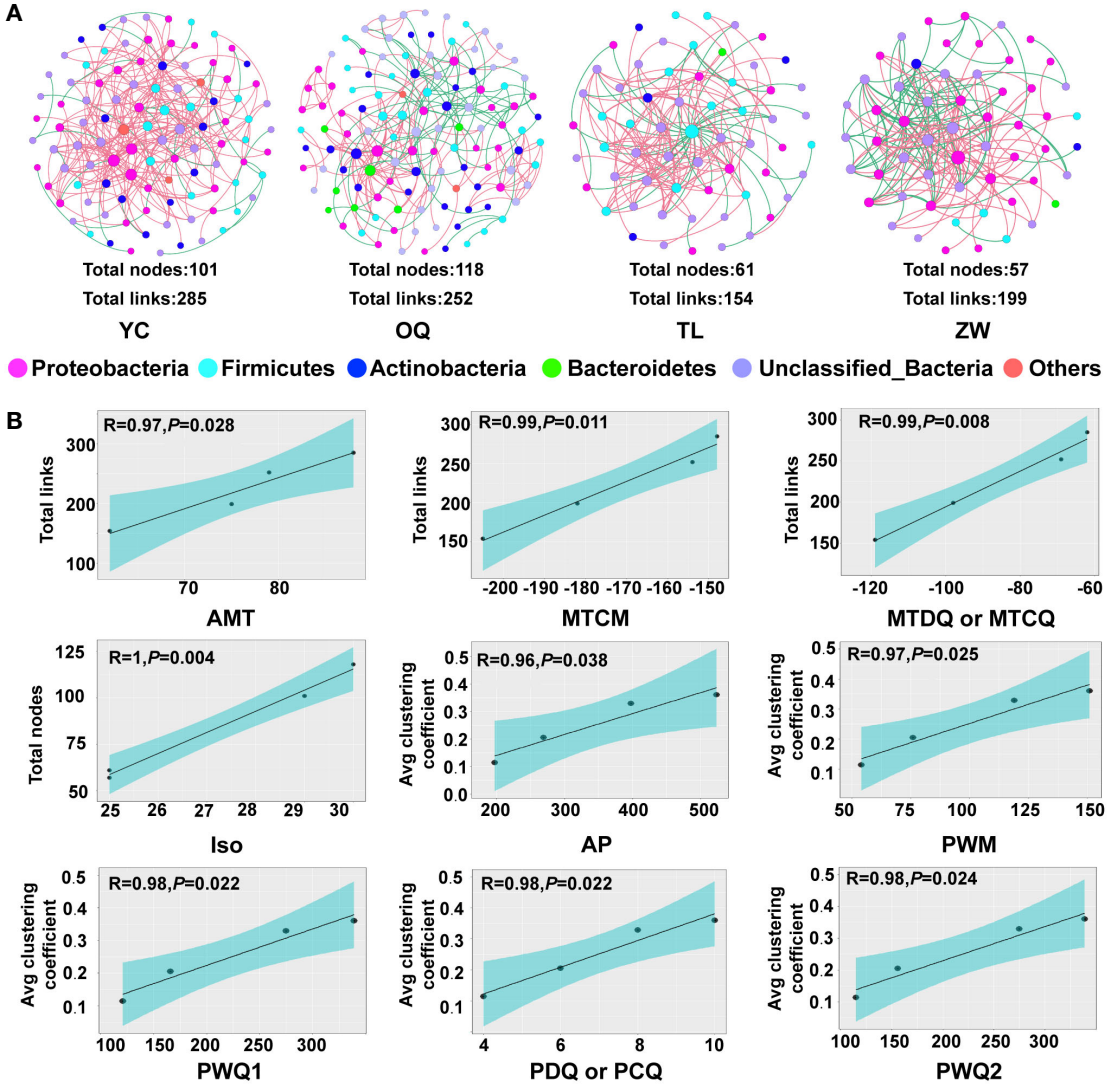
Molecular ecological networks for the phyllosphere microbial communities of yellowhorn and linear regressions between their topological properties and climatic variables. **(A)** Molecular ecological networks of phyllosphere microbial communities at the regional level. Each node indicates an amplicon sequence variant (ASV), whose size was proportional to the relative abundance of each ASV. The same colored nodes represent the same phylum, while pink or green links respective denote positive or negative correlations. **(B)** Linear regressions between topological property indices and climatic variables. Only significant relationships are shown (*P* < 0.05), the shading represents 95% confidence intervals.

The random matrix theory (RMT)-based networks were constructed to reveal the interactions among species of the phyllosphere bacterial community at the regional level. All empirical networks had higher modularity and a greater average path distance than their corresponding randomized networks, indicating the molecular ecological networks could have modularized features and small-world behavior (**Supplementary Table 7**) (Deng et al., 2012). The network topologies were described well by the power-law model (R^2^ range: 0.722 – 0.813), suggesting that most ASVs had only a few connections in the networks. The molecular ecological networks for the Yinchuan and Otogqianqi site regions had more nodes (101 and 118, respectively) and links (285 and 252, respectively), while the networks for the Tongliao and Zhangwu site regions contained fewer nodes (61 and 57, respectively) and links (154 and 199, respectively) (**Figure 5A**). Yinchuan’s bacterial network had the highest number of total links and greater average connectivity (avgK), suggesting it was more complex than the other three site region networks, especially than that of Tongliao which was relatively simple. Among all samples, the average clustering coefficient of these networks was largest for Zhangwu, albeit with relatively lower modularity. The average path length was shortest in the bacterial network for Tongliao, and longest for Otogqianqi (**Supplementary Table 7**). Taken together, the molecular ecological networks of phyllosphere bacterial communities from different regions evidently varied and could form different “small world” topological structures.

A linear regression analysis was used to reveal the major climatic variables shaping the topological structure of the phyllosphere molecular ecological networks. These results suggested the nodes, links, and average clustering coefficient of molecular ecological networks had significant linear relationships with the 13 climatic variables (**Figure 5B** **;** **Supplementary Table 8**). The numbers of nodes and links in all bacterial communities, this denoting the complexity of network properties, increased significantly with most of the temperature variables, namely, annual mean temperature, min temperature of coldest month, mean temperature of driest quarter, mean temperature of coldest quarter, and isothermality (**Figure 5B**). Besides, stronger positive associations were found between the average clustering coefficient and precipitation variables, namely, annual precipitation, precipitation of wettest month, precipitation of wettest quarter, precipitation of driest quarter, precipitation of warmest quarter, and precipitation of coldest quarter. All in all, the above-mentioned climatic variables were the most pivotal factors affecting the formation of the topological structure of the phyllosphere molecular ecological networks.

### 3.5 Keystone taxa of phyllosphere bacterial communities

**Figure 6:**
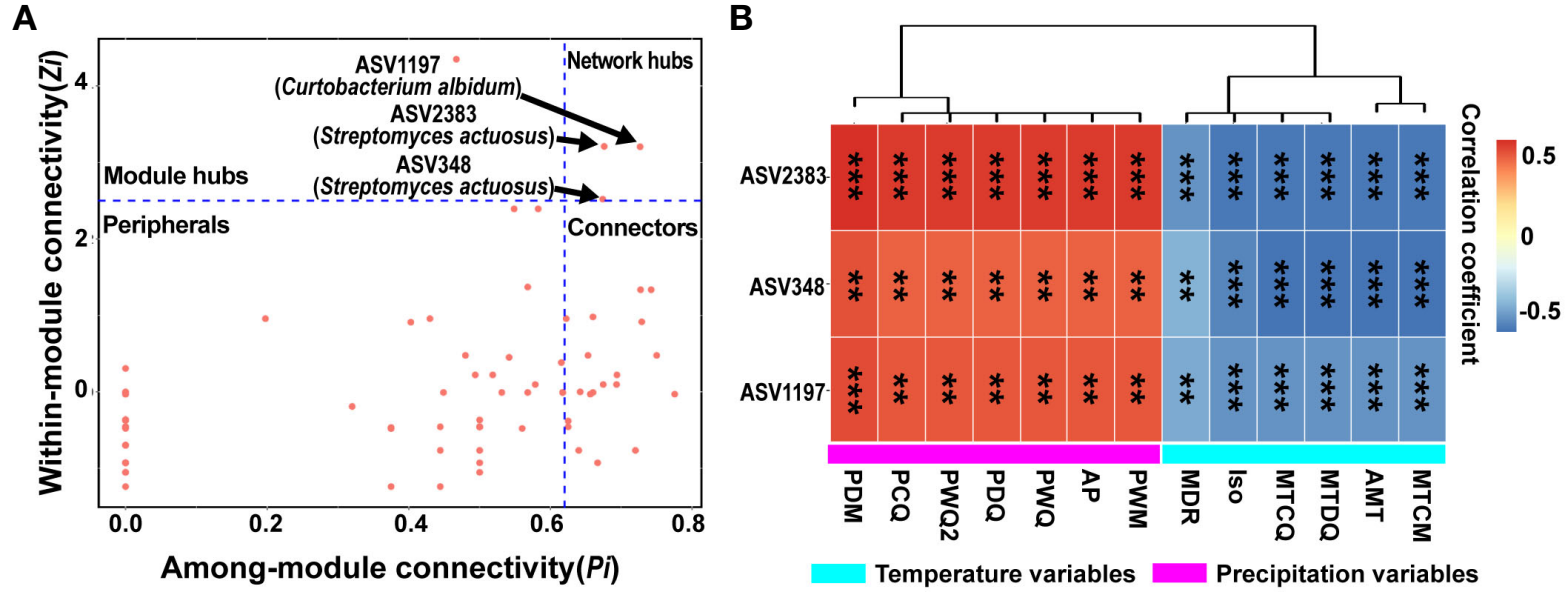
Identification of keystone taxa and correlations between these species and climatic variables. **(A)** Distribution of ASVs (amplicon sequence variants) in the overall phyllosphere microbial network based on the *Zi*-*Pi* diagram. Each symbol denotes an ASV in the network. The topological role of each ASV was determined according to the scatter plot of within-module connectivity (*Zi*) and among-module connectivity (*Pi*). Modules hubs have a value of *Zi* > 2.5, whereas connectors have a value of *Pi* > 0.62. **(B)** Correlations between keystone species and climatic variables. The rectangle’s color indicates the direction of the correlation. Asterisks indicate significant correlations between these ASVs and climatic variables (**P< 0.01; ***P < 0.001).

To identify the keystone taxa in the bacterial network, constructed using all ASVs of four sites, for the phyllosphere of yellowhorn, the distribution of ASVs in this network was evaluated using within-module connectivity (*Zi*) and among-module connectivity (*Pi*) values (**Supplementary Tables 9, 10**). All ASVs (92) in the overall network could be classified into four subcategories: network hubs (3), module hub (1), connectors (22), and peripherals (66) (**Figure 6A**; **Supplementary Table 10**). Among these important microbial species in the network’s structure, connectors were far more numerous than either network hubs or module hubs. A total of three network hubs (ASV2383, ASV348 and ASV1197) as keystone taxa belonged to the genera *Curtobacterium* and *Streptomyces* from the phylum Actinobacteria (**Figure 6A**; **Supplementary Table 10**). Another 21 ASVs were assigned to the genera *Paenibacillus*, *Herbaspirillum*, *Gillisia*, *Modestobacter*, *Bacillus*, *Natronomonas*, *Corynebacterium*, *Cellulomonas*, *Brevundimonas*, *Salinicoccus*, *Methylobacterium*, *Yaniella*, and an unclassified bacterium in the connectors (**Supplementary Table 10**). Only one module hub was identified to the genus *Massilia*.

Associations of 13 climatic variables with three network hubs were assessed further. In the overall bacterial networks, all three network hubs were significantly positively correlated with precipitation variables, but negatively correlated with temperature variables; significant associations existed between the three network hubs and all variables (**Figure 6B**). These ASVs directly connected with three network hubs were further investigated, which revealed that 21 ASVs had significant relationships with 13 climatic variables. According to Spearman correlations, significant positive relationships existed between 21 ASVs and temperature variables, whereas the correlations were negative with precipitation variables (**Figure S2**; **Supplementary Table 11**). Collectively, these temperature and precipitation variables could significantly influence the community structure of keystone taxa and their neighbors.

## 4 Discussion

It is increasingly recognized that phyllosphere bacterial communities can exert an important impact on the growth dynamics and ecological services of their host plants. Climatic conditions as a class of abiotic factors can shape the unique phyllosphere microbiota of host plant species and drive variation in the composition and structure of their phyllosphere epiphytic communities. In this field study, we found that the compositional characterization and diversity of the phyllosphere bacterial communities of yellowhorn trees differed greatly under different climatic conditions across the four site regions sampled in Northern China. This finding provides an insight perspective for revealing the pertinent regional climatic factors that play a critical role in shaping the structure of yellowhorn’s phyllosphere bacterial community.

The α- and β-diversity indices of the phyllosphere bacterial community of yellowhorn tree hosts growing in four site regions—Yinchuan, Otogqianqi, Tongliao, and Zhangwu—were examined for their dependence on 13 climatic factors. Whether gauged by the Observed species, Shannon, Evenness, or Faith_pd indice, the epiphytic phyllosphere bacterial community always significantly higher in Yinchuan than either the Tongliao or Zhangwu site regions. That higher richness, diversity, evenness, and phylogenetic diversity indices of the phyllosphere bacterial community suggests the colonization of more microbial species on the surface of leaves (Rico et al., 2014). All of the precipitation variables had significantly negative correlations with α-diversity indices, which indicated that the phyllosphere bacterial community of plants inhabiting wetter regions harbored lower diversity. This result is consistent with the idea that precipitation could remove epiphytic microbes from the leaf surface, thereby interfering with microbial colonization of leaf surfaces (Stone and Jackson, 2019). By contrast, in general the α-diversity indices of the phyllosphere bacterial communities of yellowhorn were positively correlated with the temperature variables. Warm temperature is supposed to affect the availability of carbon sources in the phyllosphere *via* direct or induced shifts in host plant metabolism, which could be a key factor underpinning changes we detected in the diversity of the phyllosphere bacterial community (Wu et al., 2011; Campisano et al., 2017; Aydogan et al., 2018). There was a distinct discrepancy between phyllosphere bacterial communities across the different site regions, clearly indicating that climatic variables can influence beta-diversity. On the whole, the phyllosphere bacterial diversity was markedly associated with the two sets of climatic factors, implying that temperature and precipitation variables are likely important for shaping the phyllosphere epiphytic bacterial community of woody plants (Al Ashhab et al., 2021).

Not only could the differences found among phyllosphere bacterial communities of yellowhorn lead to the diversity in taxa found, but they also could lead to shifts in their community composition in terms of its dominant members. The significant effects of climatic variables on phyllosphere bacterial community composition were also confirmed by the correlation analysis, which uncovered close relationships between phyllosphere bacterial abundance and climatic factors (**Figure 3**). The structural characteristics of phyllosphere bacterial communities from yellowhorn trees inhabiting the Yinchuan, Otogqianqi, Tongliao, and Zhangwu site regions were gleaned under the impact of temperature and precipitation variables. Members of the phyla Proteobacteria, Firmicutes, Actinobacteria, and Bacteroidetes predominated in the phyllosphere bacterial communities of yellowhorn in all four site regions. Proteobacteria, a high-frequency occurring phylum, was dominant in these phyllosphere bacterial communities, a finding also reported for the phyllosphere bacterial communities of other plant species (Knief et al., 2012; Aydogan et al., 2018; Roman-Reyna et al., 2019; Sahu et al., 2022). Some taxa, including *Bacillus*, *Arthrobacter*, *Carsonella*, *Ochrobactum*, *Pseudomonas*, *Candidatus Phlomobacter*, *Massilia*, and *Microbiospora*, were among the most dominant genera of the phyllosphere bacterial communities of yellowhorn, but their relative abundances differed among site regions. Previous work has found that some taxa, being likely common phyllosphere residents, can frequently occur in the phyllospheres of plant species, implying that a potential beneficial relationship could be built between these microbial species and plant hosts (Kim et al., 2012). For instance, aided by its flagellar motility, *Pseudomonas* can arrive at more favorable leaf sites, after which it can synthesize biosurfactant osmoprotectants to increase the water availability on the leaves and promote colonization on the leaf surface (Legein et al., 2020). In general, by identifying their taxonomy and members’ function, phyllosphere microbial communities can survive and bolster plant health under unstable climatic conditions, which is paramount for assembling a resilient phyllosphere system hosted by yellowhorn species.

In this study, 48 shared ASVs annotated to identified 27genera can be inferred as the core phyllosphere microbiome, whose compositional characteristics and distribution may be unique to yellowhorn trees. This core phyllosphere microbiome together represented a large proportion of the total phyllosphere community from each site region, possibly driven by the specific selection of the host plant or the adaptability of these microbial species to phyllosphere habitats (Noble et al., 2020). Some members of this core microbiome have often been found in the phyllosphere of other plant species (Cernava et al., 2019; Noble et al., 2020; Kumar et al., 2021; Sahu et al., 2022). It is known that multiple members subordinated to the core genera identified for the phyllosphere microbiome could be closely associated with host plants (Eyre et al., 2019). For example, the genus *Methylobacterium*, an indigenous member of yellowhorn’s phyllosphere microbiome, can synthesize signal molecules or compounds to help bacteria withstand the harsh environment (Lv et al., 2013; Liang et al., 2019) and can protect the host plant against damage from UV radiation (Yoshida et al., 2017). Members of the genus *Arthrobacter* can contribute to improving epiphytic fitness in the phyllosphere of host plants by modulating their gene expression (Scheublin and Leveau, 2013; Scheublin et al., 2014). Members of the Enterobacteriaceae family, being common inhabitants of phyllosphere bacterial community, are likely to occupy suitable niches and adapt to variable circumstances, due to their physiological and functional diversity (Ellis et al., 1999; Kang et al., 2015; Asaf et al., 2017). *Bacillus* and *Pseudomonas* species, as particularly familiar phyllosphere dwellers, can also promote plant growth due to their anti-phytopathogen functions (Mikiciński et al., 2015; Fessia et al., 2022). Further, apparent specific associations also emerged between some members of these core species and the climatic variables. It has been demonstrated that the relative abundances of the genera *Clostridium* and *Turicibacter* are positively correlated with temperature (Yan et al., 2021), which is in line with the results of our study. Alongside core microbiomes, non-core epiphytic phyllosphere microbial communities across the four site regions displayed obvious changes largely driven by climatic environmental factors (Noble et al., 2020) An intimate association between the host plant and specific microbial communities further indicates that these microbial groups may be functionally worthwhile for ensuring stable maintenance of the phyllosphere microbiome of a host plant (Shade and Handelsman, 2012; Noble et al., 2020).

Interestingly, the three network hubs we found to be the most prominent keystone taxa overall in the molecular ecological networks were annotated to the phylum Actinobacteria, which are copiotrophic bacteria that could resist well harsh or resource-poor environments (Acosta-Martínez et al., 2014; Maestre et al., 2015). These network hubs belonged to low abundant taxa, indicating an important role for low-abundant microbial species in maintaining network structure and functioning in the face of environmental perturbations (Lyons and Schwartz, 2001; Tao et al., 2018). In our study, there were more connectors than module hubs in the overall network, indicating more key taxa acted to connect each module and establish the whole network, while fewer ones took effect in constructing functional modules (Newman, 2006; Zhou et al., 2010; Deng et al., 2012). In this study, the higher modularity of the molecular ecological network for the phyllosphere bacterial community from Otogqianqi could assist in maintaining functional stability and weakening the impact of environmental perturbations (Kitano, 2004). In addition, that site region’s network also had the most nodes, which could exert diverse topological roles and perform multi-functionality in the complex molecular ecological network (Guimera et al., 2007; Wagg et al., 2019); this supports the view that more complex networks of microbial communities could provide more various functions to increase microbial community stability and resiliency. Nevertheless, the higher average clustering coefficient and shorter average path length of the phyllosphere bacterial network for Zhangwu than for the other site regions suggests the relationships of phyllosphere bacterial species within the network from yellowhorn in this region is better connected. These tighter connections could help microbial communities to respond very quickly to environmental stimuli and disturbances (Zhou et al., 2010).

Climatic factors can alter the relative abundance of those keystone microbes to strongly influence the total structure of phyllosphere microbial community (Aydogan et al., 2018). In this study, all network hubs, the taxa adjacent to network hubs, as well as a portion of connectors, were significantly correlated with temperature and precipitation variables, indicating that the shift of keystone species could disturb the stability of the whole phyllosphere microbiome as conditions changed in space or time. Aydogan et al. (2018) stated that warm temperature could strongly adjust the phyllosphere microbial communities by affecting critical microbial taxa. Another study also found that climatic factors could indirectly impact the composition of phyllosphere bacterial community by modulating bacterial interactions (Li et al., 2022c). It is almost certain that local changes in climate could have an impact on the ecosystem functioning of the phyllosphere microbiome given the narrow climatic endurance of its key microbial taxa (Xing et al., 2022). Besides, the links and nodes of the phyllosphere bacterial community networks from yellowhorn had positive and negative correlative relationships with the temperature and precipitation variables, respectively. This indicates that more temperature and less precipitation were conducive for fostering phyllosphere microbial network complexity in an appropriate range. Work (Li et al., 2022c) also demonstrated that less precipitation could strengthen the interactions and cooperation between co-occurring microbial functional taxa, possibly due to greater bacterial separation and reduced competition between bacterial species under conditions of little precipitation (Treves et al., 2003). Previous studies also affirmed that appropriately higher temperatures could intensify both the network connectivity and complexity of microbial communities to improve the cooperation between member microbes to defend the impaired ecosystem functions (Yuan et al., 2021; Xing et al., 2022). Therefore, combining climatic factors and key microbial interactions could reinforce the regulation of phyllosphere bacterial community assembly on yellowhorn hosts.

It is widely accepted that the assembly of phyllosphere microbiome is considerably influenced by a few abiotic factors related to climatic conditions (Faticov et al., 2021; Bashir et al., 2022). In this study, we investigated the bacterial phyllosphere microbiome of the yellowhorn grown in four sampling sites of distinctive climatic features located across north China. Interestingly, our findings clearly display the differentiated responses of phyllosphere bacterial communities of the yellowhorn tree to precipitation and temperature regimes. However, it is important to note that, due to the absence of the gradient of temperature and precipitation of each sampling site, it is hard to determine if these differentiated responses are affected by other factors of the sampling sites, for example, application of pesticides and foliar fertilizer (Vacher et al., 2016; Faticov et al., 2021), or how much those factors influence these contrary responses. To clarify this, we will select and set more sampling sites in each of the four sampling site regions (Yinchuan, Otogqianqi, Tongliao and Zhangwu) across north China based on their climate data to build the gradients of temperature and precipitation of each sampling site region or establish gradients of temperature and humidity in the controlled conditions of greenhouse or laboratory to better interpretate the correlation between the yellowhorn phyllosphere bacterial communities and climatic variables in our future work. Furthermore, the reductionist synthetic community (RSC) method based on microbial cultivation (Niu et al., 2017; Niu et al., 2020; Wu et al., 2023), together with the plant phenomics technology approach (Roitsch et al., 2022; Zavafer et al., 2023), may be employed to validate the opposite responses to rainfall and temperature.

## 5 Conclusions

In this study, we investigated the structural characteristics of natural phyllosphere bacterial communities of yellowhorn trees across multiple regions in the temperate zone of China and the climatic factors impacting their distribution. We show that temperature and precipitation variables exert influence on the composition, diversity, and network properties of phyllosphere bacterial communities that occur on yellowhorn leaves. Temperature variables mainly have positive correlations with the core species, network hubs, most ASVs, and the α-diversity indices, whereas the precipitation variables are negatively correlated with them. We found many core species extant in the overall molecular ecological network, suggesting their close relationship to network taxa within phyllosphere epiphytic bacterial communities, which in turn plays an important role in maintaining bacterial community stability and functionality. In addition, network complexity of the phyllosphere bacterial community is positively correlated with temperature, indicating that a higher temperature could help to maintain functional stability and weaken the impact of environmental perturbations. Taken together, these findings broaden our current understanding of climate-driven processes for establishing phyllosphere bacterial communities of yellowhorn in its four main production regions in China, with important implications for predicting their eco-evolutionary dynamics under ongoing climate change.

## Data availability statement

The original contributions presented in the study are included in the article/**Supplementary Material**. Further inquiries can be directed to the corresponding authors.

## Author contributions

WW: Writing – review & editing, Methodology, Writing – original draft, Data curation, Visualization. CH: Methodology, Visualization, Writing – original draft. YC: Methodology, Investigation, Writing – original draft. LW: Resources, Writing – original draft. QB: Resources, Writing – original draft. XL: Resources, Writing – original draft. ZZ: Resources, Writing – original draft. XZ: Funding acquisition, Methodology, Writing – review & editing. DW: Funding acquisition, Methodology, Writing – review & editing. BN: Funding acquisition, Methodology, Project administration, Writing – review & editing, Writing – original draft.
