# Supplementary material for: Differentiated responses of the phyllosphere bacterial community of the yellowhorn tree to precipitation and temperature regimes across Northern China
Source: Front Plant Sci. 2023 Oct 25;14:1265362. doi: 10.3389/fpls.2023.1265362 (PMC10634255; doi:10.3389/fpls.2023.1265362)

Differentiated responses of the phyllosphere bacterial community of the yellowhorn tree to precipitation and temperature regimes across Northern China

**Weixiong Wang^1,2,3^, Congcong Hu^4^, Yu Chang^1, 3^, Libing Wang^5^, Quanxin Bi^5^, Xin Lu^6,7^, Zhimin Zheng^1,2^,** **Xiaoqi Zheng^8*^, Di Wu^1,2,3*^ and Ben Niu^1,2,3*^**

^1^State Key Laboratory of Tree Genetics and Breeding, Northeast Forestry University, Harbin, China

^2^The Center for Basic Forestry Research, College of Forestry, Northeast Forestry University, Harbin, China

^3^College of Life Science, Northeast Forestry University, Harbin, China

^4^Department of Mathematics, Shanghai Normal University, Shanghai, China

^5^State Key Laboratory of Tree Genetics and Breeding, Research Institute of Forestry, Chinese Academy of Forestry, Beijing, China

^6^Chifeng Research Institute of Forestry Science, Chifeng, China

^7^National Forestry and Grassland Shiny-Leaved Yellowhorn Engineering and Technology Research Center, Chifeng, China

^8^Center for Single-Cell Omics, School of Public Health, Shanghai Jiao Tong University School of Medicine, Shanghai, China

*** Correspondence:**

Ben Niu

e-mail: ben_niu@nefu.edu.cn

Di Wu

e-mail: wudi_nefu@nefu.edu.cn

Xiaoqi Zheng

e-mail: xqzheng@shnu.edu.cn

**
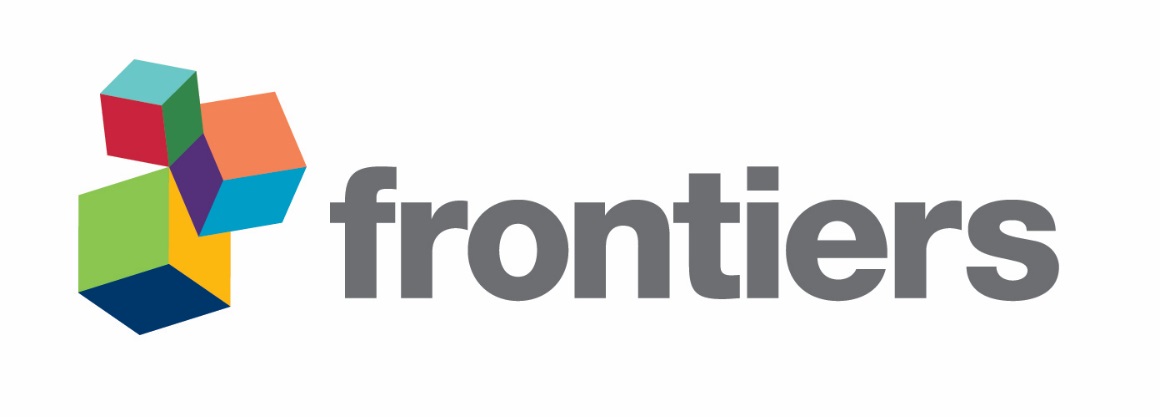
**

**Supplementary FIGURE 1 Geographical locations of four sampling sites.** Diamonds of different colors on the map represent different sampling sites. YC, Yinchuan; OQ, Otogqianqi; TL, Tongliao; ZW, Zhangwu. Scale: 1:100000000.

**Supplementary FIGURE 2** **Correlations between ASVs directly connected with three network hubs and climatic variables**. The orange color denotes a positive correlation, while the green color denotes a negative correlation. Asterisks indicate significant correlations between ASVs directly connected with three network hubs and climatic variables (* *P* < 0.05; ** *P*< 0.01; *** *P*< 0.001).

**
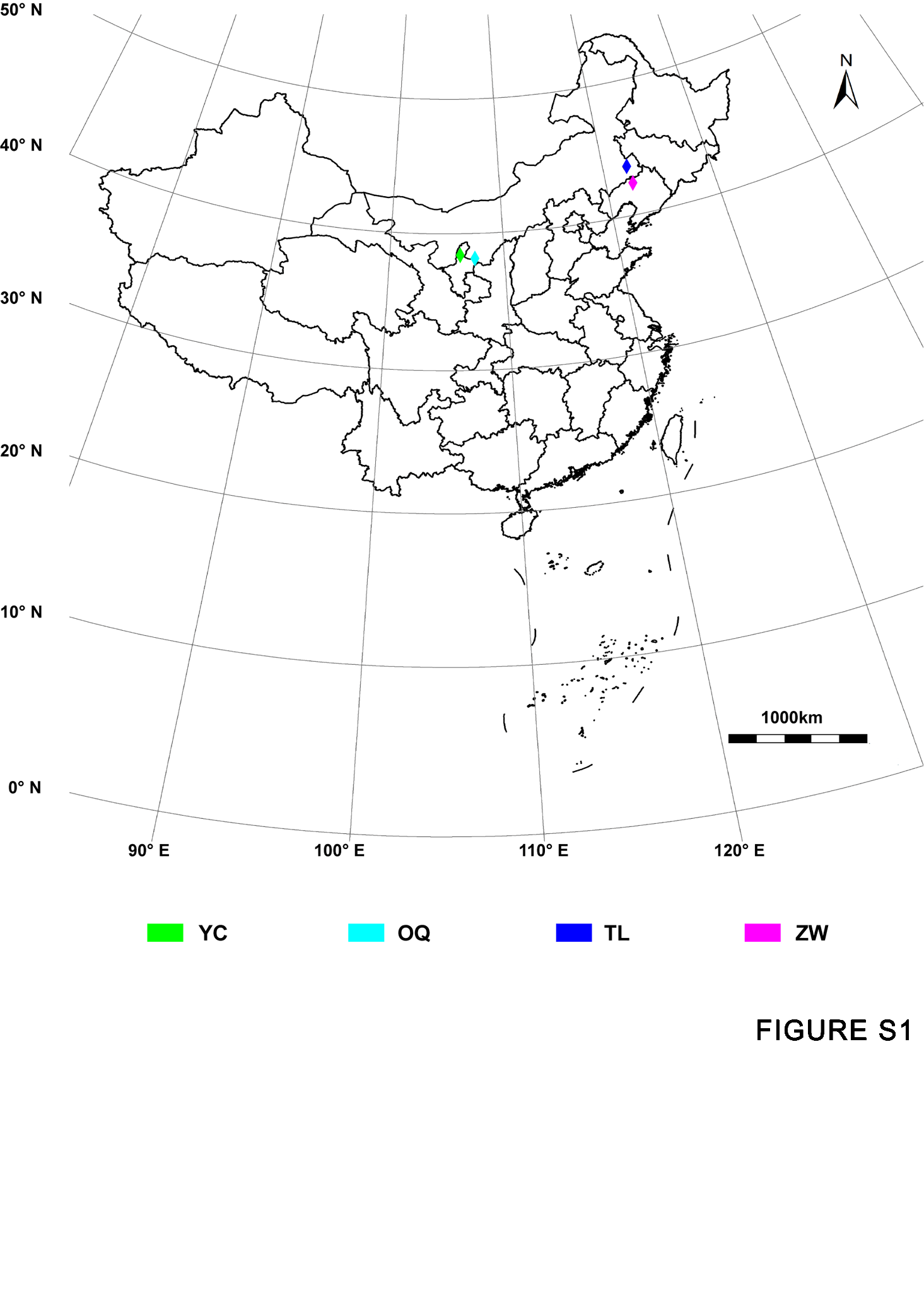
**


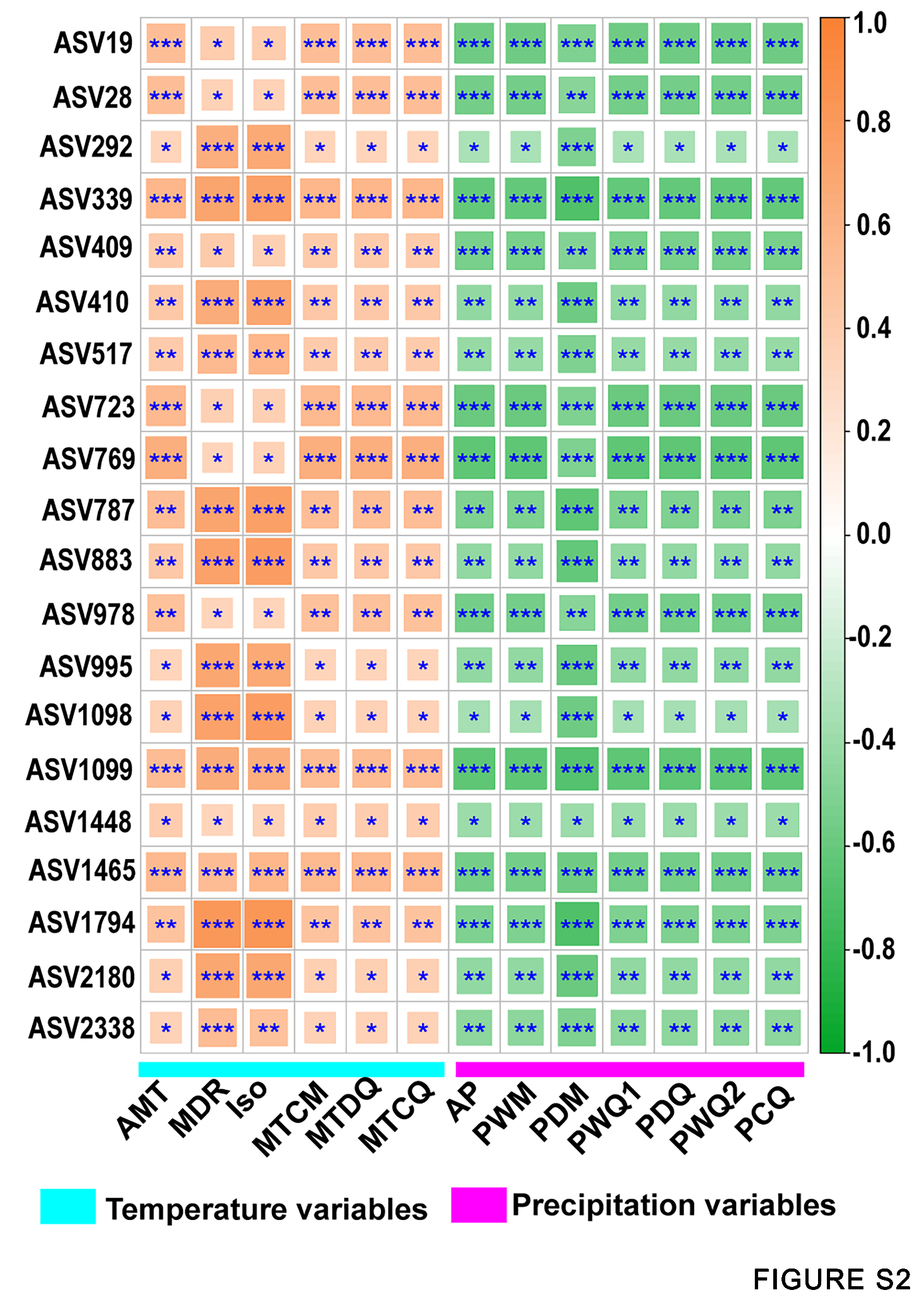

Supplement: Supplementary file 1 [file DataSheet_1.zip › Supplementary Figures.docx]
